# Epidemiological analysis of stroke patients with emphasis on access to acute-phase therapies

**DOI:** 10.1590/0004-282X-ANP-2020-0466

**Published:** 2022-02-06

**Authors:** Camila Favoreto do ROSÁRIO, Walker Garcia FERNANDES, André Luiz PESSOTTI, Beatriz Cardoso RODRIGUES, Juliana Diniz BAPTISTA, Marcela SEGATTO, Vinicius Santana NUNES, Leandro de Assis BARBOSA, Abraão Ferraz Alves PEREIRA, Christiane Lourenço MOTA, José Antônio FIOROT

**Affiliations:** 1 Faculdade Brasileira Multivix, Vitória ES, Brazil. Faculdade Brasileira Multivix Vitória ES Brazil

**Keywords:** Stroke, Mechanical Thrombolysis, Health Profile, Public Health, Acidente Vascular Cerebral, Trombólise Mecânica, Perfil de Saúde, Saúde Pública

## Abstract

**Background:**

Stroke is a public health problem. For patients with ischemic stroke, venous thrombolysis and mechanical thrombectomy are effective therapeutic options. However, even after the National Stroke Treatment Guidelines were published in 2012, the number of cases treated is still lower than expected.

**Objective:**

To identify the determining factors for obtaining access to acute-phase therapies in the state of Espírito Santo (ES) and investigate the profile of stroke patients treated at the Central State Hospital (HEC).

**Methods:**

Retrospective data from the medical records of 1078 patients from May 2018 to December 2019 were analyzed.

**Results:**

Among the 1,078 patients, 54.9% were men and the most prevalent age group was 60 to 79 years. Systemic arterial hypertension was the main single risk factor. Regarding treatment modality among the patients who arrived at the HEC within the therapeutic window, 47% received some type of acute-phase therapy. Waking up with the deficit was the main contraindication for venous thrombolysis in these cases.

**Conclusions:**

Application of the flowchart established by SESA-ES seemed to be effective for enabling responsiveness of care for stroke victims. Public emergency transport services had a fundamental role in this process. In addition, the care provided by the tertiary stroke center provided excellent access to acute-phase therapies. However, despite the efficiency of the service provided at the HEC, it only reached a maximum of 50% of the ES population. This service model therefore needs to be expanded throughout the state.

## RESUMO

**Antecedentes:**

O acidente vascular cerebral (AVC) é um problema de saúde pública. Nos casos de AVC isquêmico, a trombólise venosa e a trombectomia mecânica são efetivas opções terapêuticas de fase aguda. Entretanto, mesmo com a Diretriz Nacional de AVC publicada desde 2012, o número de casos tratados ainda é baixo.

**Objetivo:**

Apurar os fatores determinantes para o acesso às terapias de fase aguda na realidade espírito-santense e investigar o perfil dos pacientes de AVC atendidos no Hospital Estadual Central de Vitória (HEC).

**Métodos:**

O presente estudo analisou dados retrospectivos de prontuários de 1.078 pacientes no período de maio de 2018 a dezembro de 2019.

**Resultados:**

Dos 1.078 pacientes, 54,9% eram homens e a faixa etária mais prevalente foi a de 60 a 79 anos. A hipertensão arterial sistêmica foi o principal fator de risco isolado. Quanto ao tratamento, identificou-se que entre os pacientes que chegaram ao HEC na janela terapêutica 47% receberam terapia de fase aguda e que acordar com o déficit foi a principal contraindicação para trombólise venosa nesses casos.

**Conclusões:**

As análises demonstraram que a aplicação do fluxograma estabelecido pela Secretaria de Estado da Saúde do Espírito Santo parece ser eficaz na agilidade de atendimento das vítimas de AVC e que o Serviço de Atendimento Móvel de Urgência tem um papel fundamental nesse processo. Além disso, a assistência de um centro terciário de AVC permite acesso às terapias de fase aguda com excelência. Todavia, mesmo que o modelo de serviço prestado no HEC seja eficiente, ele atinge no máximo 50% da população do ES, sendo necessária a sua ampliação.

## INTRODUCTION

Stroke is an important public health problem and has been the second leading cause of death worldwide since the year 2000^1^. In Brazil, the prevalence of stroke is no different, since in 2017 it was the third leading cause of death. In the state of Espírito Santo, the number of occurrences is also significant, with more than 4,000 cases of hospitalization for stroke per year^2^. According to the World Health Organization, stroke is defined as the development of sudden neurological deficits, resulting from a vascular injury, with symptoms lasting 24 hours or more, which compromises cognitive, sensory or motor functions^3^. Most of the patients who survive have permanent cognitive and motor sequelae, which impacts family and social dynamics^4^.

Among the therapeutic possibilities for ischemic stroke, venous thrombolysis (VT) is one of the most effective. It can increase the chances of a good clinical outcome by up to 30%^5^. VT is currently indicated for patients with evolution of up to 4.5 hours from the time of symptom onset (ictus), while respecting inclusion and exclusion criteria^6^. This form of treatment was approved in Brazil in 2001 but, despite recent advances in this treatment class, the number of patients who have access to it is still low in this country^7^. Mechanical thrombectomy (MT) is another acute-phase treatment that has been established for stroke. Its use has been correlated with a chance more than 50% of reducing functional impairment^8^. It has been indicated for patients who present arterial occlusion of proximal vessels and with up to 6 hours of ictus, while also respecting clinical and radiological criteria^9^.

Given that the characteristics of stroke are severe, it is clear that urgent adequate medical assistance needs to be sought as soon as the first signs and symptoms appear. Time is a critical component for enabling access to the existing therapies^10^.

In order to standardize patient care in the most diverse regions of Brazil, the Ministry of Health made stroke care guidelines available in 2013^11^. These provide instructions for professionals at all levels of healthcare. Following this same logic, in 2018 the Health Department of the State of Espírito Santo (SESA-ES) published the Clinical Guidelines for Managing Stroke Patients. Through this document, it was sought to reduce morbidity and mortality due to stroke. A more specific flow for care, referring to service units, was described^12^.

The aims of this study were to investigate the profile of stroke victims treated at the Central State Hospital (HEC) in Vitória, from May 2018 to December 2019, and to examine the determinant factors for achieving access to acute-phase therapies within the realities of the state of Espírito Santo.

## METHODS

This study was carried out after a consent statement had been signed by the board of HEC and approval had been obtained from the Institutional Review Boards (IRB) of the Multivix Faculty of Teaching, Research and Extension.

To develop this study, secondary data obtained retrospectively through analysis of the contents of medical records were evaluated. The study included all patients who were referred to HEC with suspected stroke, from May 2018 to December 2019, as long as they were 18 years old or older and had presented ictus within less than 6 hours. Patients with ictus up to 24 hours were also included, as long as the Glasgow Coma Scale score was greater than 10. Patients who had diagnoses of hemorrhagic stroke with surgical indication, subarachnoid hemorrhage (SAH), brain tumors, neuroinfectious or malignant middle cerebral artery (MCA) stroke with an indication for urgent surgery were excluded from the analyses.

The following data from the medical records were analyzed: sociodemographic data; patient’s place of origin; time interval between ictus and hospital admission (called ictus-to-admission time); time interval between telephone contact from SAMU (public emergency transport service) to the hospital and hospital admission (called SAMU-to-admission time); presence of risk factors for stroke; number of patients undergoing acute-phase therapies (VT and/or MT); time interval between the onset of symptoms and the start of acute-phase therapy (called the therapeutic window); and time interval between hospital admission and infusion of the bolus of the thrombolytics (called door-to-needle time, DNT).

After data collection, statistical analyses were performed using the SPSS software version 24.0. Asymmetries were observed with regard to the continuous descriptive variables according to the Shapiro-Wilk test (p<0.05) and these were therefore taken as the median, minimum and maximum and 95% confidence interval (95%CI). Nominal variables were presented as frequencies and percentages. To check the influence of sex, age and place of origin on the therapeutic window, the chi-square test was used; and on the ictus-to-admission time, the Mann-Whitney and Kruskal-Wallis nonparametric tests were used. The significance level was taken to be 0.05. Cross-validations, Student’s *t*-tests and ANOVA allowed possible correlations between the variables analyzed to be determined and conclusions to be reached.

## RESULTS

**Figure 1.**
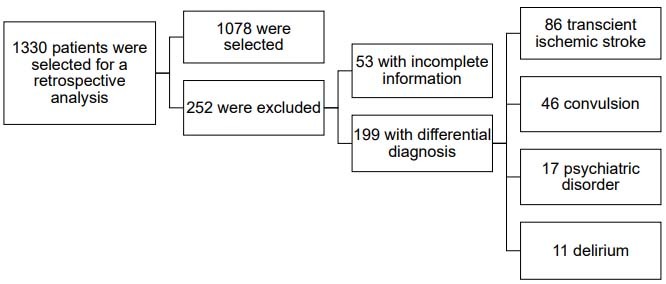
Patients selected for the study, according to the inclusion and exclusion criteria.

Retrospective data from 1,330 patients were evaluated, as shown in the flowchart in Figure 1.

**Figure 2.**
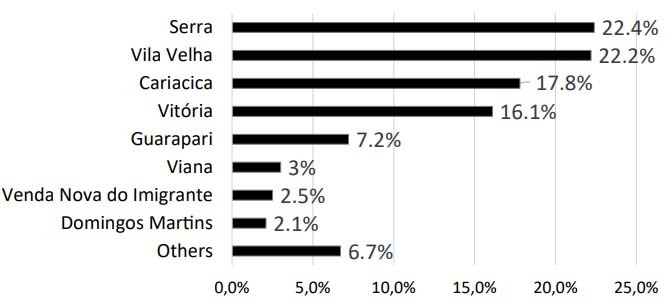
Municipality of origin and percentages of the total number of patients treated.

Regarding the sociodemographic profile, the patients’ ages ranged from 20 to 98 years, with a median of 67 years. The data were thus divided into three age categories: young, patients aged between 20 and 59 years; middle-aged, 60 to 79 years old; and extremely aged, 80 years old or over. Middle-aged was the most prevalent category, accounting for 74.3% of the cases, followed by extremely aged with 16.5% and, lastly, the young category with only 9.2%. Regarding sex, men were more affected by stroke, with 54.9% of the cases. Also within the sociodemographic profile, it was possible to demonstrate that the HEC received patients from 18 different cities, distributed as shown in Figure 2.

Clinically, the patients were assessed for isolated risk factors that may have been present in their clinical history. Systemic arterial hypertension was found to be present in 75% of patients and was the most prevalent factor, followed by diabetes in 31% of the cases, previous stroke in 21.5% and smoking in 21%. Less than 1% of the patients had no risk factors. The most prevalent type of stroke among the patients assisted was ischemic stroke, which was seen in 97.8% of the sample. This very high rate of stroke was expected, considering the inclusion and exclusion criteria of the study.

The profile of the healthcare provided at the HEC was also evaluated using different variables. The patients’ place of origin was registered, as shown in Table 1.

**Table 1.** Place of origin of patients treated at Central State Hospital, with respective percentages.

Place of origin (n=1,078)	n (%)
Emergency room	424 (39.3)
Household	355 (32.9)
GV public hospital	141 (13.1)
State inland hospital	70 (6.5)
Others	44 (4.1)
Public road^*^	25 (2.3)
GV private hospital	19 (1.8)

GV: Greater Vitória; *Rescued by SAMU on public roads.

Ictus-to-admission time was registered retrospectively for only a portion of our sample (n=729 patients), as these data were not available in some medical records that were evaluated. This time was divided into different categories according to the therapeutic window for acute-phase treatment, and is shown in Table 2.

**Table 2.** Ictus-admission time in hours (n=729).

Ictus-admission time in hours (n=729)	n (%)	Median (min-max)	(95%CI)
Up to 1 hour	20 (2.7)	212 (8-1,800)	(201.5-228)
Between 1 and 3 hours	287 (39.4)		
Between 3 and 4.5 hours	168 (23)		
Between 4.5 and 8 hours	172 (23.6)		
More than 8 hours	82 (11.2)		

95%CI: 95% confidence interval.

Table 3 summarizes the main data relating to the temporality of the care provided. Door-to-needle time was evaluated only for patients who underwent thrombolytic treatment.

**Table 3.** Median (min-max) of SAMU-admission time and door-to-needle time, in minutes, among patients seen at Central State Hospital (95%CI).

Median of SAMU-admission time and door-to-needle time, in minutes, among patients seen at HEC	Median (min-max)	95%CI
SAMU-admission contact (n=1,047)	56 (5-420)	54-59
Door-to-needle in minutes (n=294)	28 (0-86)	27-31

SAMU: Public Emergency Transport Service; HEC: Central State Hospital; 95%CI: 95% confidence interval.

**Figure 3.**
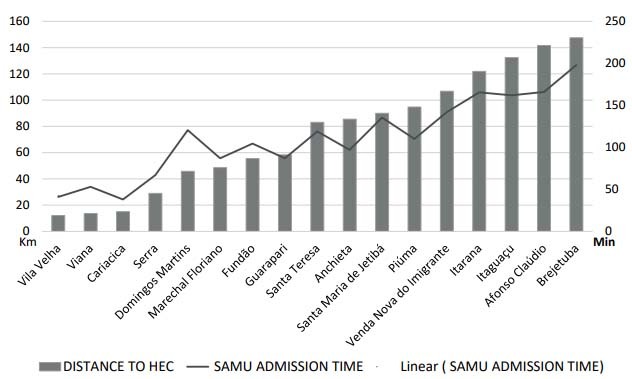
Relationship between SAMU-to-admission time and municipalities from which the patients treated at the Central State Hospital came.

HEC: Central State Hospital; SAMU: Public Emergency Transport Service.

The analysis on the SAMU-to-admission time was extrapolated to ascertain any possible correlations with the distance from the city of origin to the HEC, as shown in Figure 3.

The ictus-to-admission time was also detailed with a view to searching for possible correlations with gender, age and place of origin, as shown in Table 4.

**Table 4.** Relationship between ictus-admission time and the variables of gender, age and place of origin, indicating the median value for each situation (min-max) and (95%CI).

		Ictus-to-admission time (in minutes)	Median (95% CI)	p-value
Gender (n=729)	Male (n=405)	218 (12-1,440)	194-234	0.875
	Female (n=324)	210 (8-1,800)	191-225	
Age (n=729)	Young	205 (24-1,440)	159-240	0.40
	Middle-aged	219 (12-1,800)	201-231	
	Extremely elderly	204 (8-1,320)	172-245	
Place of origin (n=602)*	Residence	214 (30-1,140)	182-240	0.756^a^
	Healthcare centers	212 (8-1,140)	197.7-229.5	
	Spontaneous demand	295 (60-960)	107-540	

95%CI: 95% confidence interval; *Greater Vitória municipalities only; ^a^Kruskal-Wallis test.

**Figure 4.**
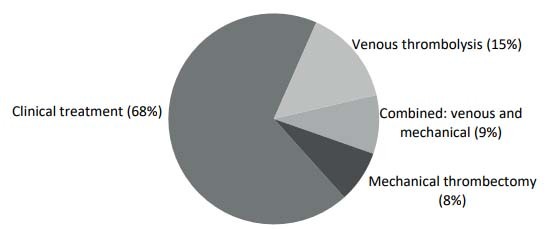
Percentages of treatments, according to the categories of therapeutic modalities.

After evaluation, the patients were guided to undergo one of the therapeutic modalities thus categorized: clinical treatment, for patients who received anticoagulation, anti-aggregation and/or statin; venous thrombolysis; mechanical thrombectomy; or combined therapy for patients who underwent venous thrombolysis and mechanical thrombectomy. Clinical treatment was the most prevalent modality (68.3%) (Figure 4).

Also with regard to treatment modality, it was found that among the patients who managed to arrive at the HEC within the therapeutic window of up to 4.5 hours, 47% received acute-phase treatment. Table 5 shows the main reasons why patients who arrived within the therapeutic window did not undergo venous thrombolysis (VT) interventions. Table 5 also shows the main reasons for delays in VT procedures, in cases in which such delays occurred.

**Table 5.** Reason for not applying venous thrombolysis and reasons for delayed venous thrombolysis procedures.

		n (%)
Contraindication for VT (n=177)	Wake up with deficit	39 (22)
	NIHSS low (<2)	26(14.7)
	Others	39 (22)
	Indeterminate ictus	24 (13.6)
	Contraindication on CT	26 (14.7)
Justification for delay in starting VT (n=53)	Difficulty in controlling blood pressure	27 (50.9)
	Others	19 (35.8)
	Difficulty in obtaining intravenous line	7 (13.2)
	Delay in laboratory	4 (7.5)
	Angiotomography	3 (5.7)
	Hemodynamic definition	2 (3.8)

VT: venous thrombolysis; NIHSS: National Institutes of Health Stroke Scale; CT: computed tomography.

## DISCUSSION

The state of Espírito Santo has more than 4,000 hospitalizations caused by stroke per year^2^. During the study period, 1,078 admissions due to this disease were registered at HEC. Considering that these data related to a period of about two years, and that in 2018 and 2019, HEC was the only public hospital in the state that had a stroke unit, it is possible to estimate that about 3,000 people who were hospitalized due to stroke in this state did not receive the ideal treatment^6^. Ordinance no. 665, of April 12, 2012, established financial incentives for stroke units to be set up within the scope of the Brazilian National Health System (*Sistema Único de Saúde* [SUS], in Portuguese). According to this document, professional training would be necessary for emergency care, and for expanding access to diagnostic tests and VT^13^. Pontes-Neto also highlighted the importance of effective changes in order to increase the availability of MT, in addition to reinforcing the pre-hospital environment through development of strategies that would improve the patient care process^14^. The data gathered in the present study demonstrated that stroke was more prevalent among men and middle-aged people, in agreement with the data of Locatelli et al., who found the same profile in their study^13^. However, it is known that there is a direct relationship between increased incidence of stroke and aging^14^, and the high prevalence of middle-aged patients can be explained simply through the absolute number of people in this age group in ES, which is more than five times the number of elderly people over 80 years old^15^. The patients classified as young people with stroke accounted for 9% of the sample, and this was within the range proposed by Smajlovic, who affirmed that although the proportion of young people with stroke would vary between countries, it would lie between 5 and 20%^16^.that stroke is a disease with high morbidity, it is important to note that stroke among economically active individuals directly impacts the economy of a country, which therefore emphasizes the need for research aimed at preventing stroke in this age group.

**Figure 5.**
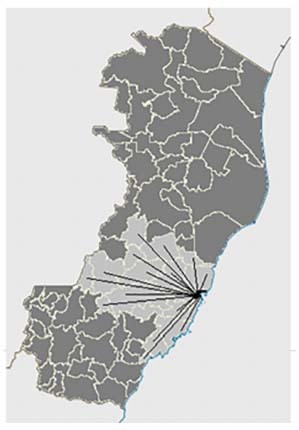
Map highlighting the municipality of Vitória, where the Central State Hospital is located, and the radius of coverage of Public Emergency Transport Service among the municipalities of Espírito Santo.

HEC received patients from 18 municipalities in ES. Serra was the city that sent the most patients, followed by Vila Velha. Together, these two cities accounted for more than 50% of the sample, which can be explained by the fact that they are the most populous cities in the state^17^. According to the IBGE (*Instituto Brasileiro de Geografia e Estatistica*, in Portuguese), ES has 78 municipalities, so our sample represented only 23% of them. The explanation for the low number of municipalities represented may be related to the areal coverage provided by SAMU during the period studied (Figure 5), since all 18 municipalities with SAMU coverage sent patients to HEC^18^. On the other hand, despite the sample only representing 23% of these municipalities, these 18 cities have more than 50% of the population across the state, reaching approximately 2 million inhabitants^17^.

Hypertension (HT) is the comorbidity that most correlates with stroke^14^^,^^19^. In the present study, 75% of the patients had HT, which made it the most prevalent risk factor among HEC patients, followed by diabetes, previous stroke and smoking. The importance of knowing the risk factors is corroborated by Passos, who stated that the incidence of stroke can be reduced through public policies aimed at knowing the etiology, risk factors and the profile of patients^20^.

According to data in the literature, patients who resorted to SAMU or went directly to the hospital were able to access specialized care more quickly^10^^,^^7^. Among the patients seen at HEC, 39.3% were referred to emergency rooms, which demonstrates the need to direct the focus of public healthcare policies towards informative work on early recognition of symptoms and the need to quickly search for a stroke reference center, preferably using the SAMU.

Time was a widely explored variable in this study, given that it is a decisive factor for the prognosis of stroke patients^21^. It was found that 67% of the patients managed to reach the HEC within the therapeutic window for VT. In a study by Panício, less than 25% of the patients arrived at the hospital within 4.5 hours^10^, which may suggest that the flowchart for care proposed by SESA-ES has good application. However, it is worth mentioning that the inclusion and exclusion criteria of our study prevented patients with ictus longer than 24 hours from being referred to the HEC, which may have overestimated the percentage of patients seen within the therapeutic window.

It was also possible to separately analyze SAMU’s performance within the service flowchart. In the correlation between SAMU-to-admission time and the distance from the city of origin to the HEC, there was a relationship with a trend towards linearity, but it had some points of variation, such as the city of Domingos Martins. The possible causes of these variations may be associated with the distance from the SAMU base, as well as the highway that formed the route taken in travelling to the hospital. It is worth mentioning that for 95% of the patients, their journey using SAMU was completed in less than 1 hour, which shows the effectiveness of SAMU for transporting these patients.

In analyzing the standard of care provided at the HEC, the door-to-needle time was observed. This demonstrated that 95% of the patients were assisted within 30 minutes. This highlights the efficiency of this specialized service for caring for stroke patients, since in the NINDS study the recommended time from admission to infusion of the thrombolytics was up to 60 minutes^21^, i.e. twice the time spent, on average, at HEC.

Delays in receiving adequate care prevent patients from benefiting from therapeutic advances that would ensure their survival or minimize the severity of their injuries^22^. In this light, the ictus-to-admission time was correlated with the factors of gender, age and place of origin, in order to identify possible differences between the groups. However, there was no statistically significant relationship between this time and the variables analyzed. It is worth mentioning that there was a tendency towards longer ictus-to-admission times for patients who arrived at the hospital as spontaneous demand, which suggests that this healthcare service was efficient regarding care chronology, when care was requested.

Although the numbers still remain high, the stroke mortality rate has been tending to decrease in Brazil,^22^. According to Pinheiro and Vianna, this scenario is due to controlling for known cardiovascular risk factors and development of new diagnostic technologies and highly complex therapeutic procedures^3^. However, in Brazil, stroke units continue to function precariously, which compromises the implementation of these therapies^23^.

Venous thrombolysis (VT) has been consolidated as a successful therapeutic option since 1995. A large study has proven the benefit of thrombolytic infusion in stroke patients^21^^,^^24^. MT has also appeared as a complementary strategy for extending the treatment of stroke. Its indication has expanded the therapeutic window to up to 6 hours after the onset of symptoms, in selected cases of occlusion of large vessels that respect the inclusion criteria of the international guidelines. In any case, time remains fundamental with regard to the results that can be obtained^14^. In the present study, acute-phase therapeutic interventions were performed on 32.44% of the stroke patients treated at HEC, which is a high rate even by international standards^25^^,^^26^. Nevertheless, this demonstrates that there is a need to expand VT and MT care, considering the efficiency of these therapies not only for reducing mortality but also for reducing the disabilities acquired by patients^27^.

In a specific analysis on patients who managed to reach HEC within the therapeutic window, it was identified that 53% of them underwent clinical treatment. The perception of neurological deficit when waking up was the most prevalent variable that prevented other patients from receiving VT. The so-called “WakeUpStroke” (WUS) treatment option is designated for this type of situation, which is not uncommon in healthcare services and constitutes a limitation to thrombolytic therapy^28^. Unfortunately, the vast majority of emergency medical services do not have magnetic resonance imaging (MRI) devices available, and this is also the case with HEC. MRI would be important because it allows identification of areas of darkness that potentially have recoverable brain tissue^26^. It is worth mentioning that some studies^28^^,^^29^ have already managed to obtain good results from MT applied within 16 and 24 hours of the ictus, which allows expansion of the therapeutic window to include the majority of patients who wake up with a deficit.

The main factors that culminated in delay to the VT procedure were difficulty in controlling blood pressure and in obtaining intravenous access. These situations reflect reports in the literature and corroborate the data from Raffin et al (2006), who stated that before the procedure begins, two peripheral venous accesses need to be obtained, through which the medication will be administered. In addition, use of thrombolytics implies the need for strict pressure management, due to the risk of correlated cerebral hemorrhage^30^.

Our study was limited by the fact that it was carried out in a single stroke center, where patients are received from only some of the municipalities in ES. Thus, our data did not portray the reality of the entire state. Furthermore, the screening criteria for patients referred to the HEC prevented broader analysis of patients with ictus longer than 24 hours.

In conclusion, stroke is a public health problem and epidemiological studies have shown the importance of preventing and treating this disease and of promoting countermeasures against stroke in Brazil.

It was found that application of the flowchart established by SESA-ES seemed to be effective for improving the responsiveness of care for stroke victims, and that SAMU had a fundamental role in this process. It was noteworthy that it is necessary to expand the coverage of the mobile emergency service in this state, in order to achieve good outcomes throughout its area.

Moreover, the importance of the care provided by this tertiary stroke center could be demonstrated through the responsiveness and efficiency of the service, in which acute-phase therapies were ensured for 32.44% of the patients with stroke. This is a very high rate, even by international standards. Therefore, it is necessary to structure other similar units in different regions of Espírito Santo.

Further studies are needed in other regions of the state in order to better characterize the reality of stroke treatment in ES. Even with the efficiency of the service provided in the HEC stroke unit, this unit reaches a maximum of 50% of the ES population, which is not representative of the entirety of the state’s healthcare service.
